# BRINGING ART TO LIFE: EXPERIENTIAL LEARNING OUTCOMES WITH GRADUATE AND UNDERGRADUATE STUDENT LEARNERS

**DOI:** 10.1093/geroni/igad104.0368

**Published:** 2023-12-21

**Authors:** Rebecca Allen, Keisha Carden, Candice Reel, Daniel Potts

**Affiliations:** The University of Alabama, Tuscaloosa, Alabama, United States; VA Maryland Health Care System, Baltimore, Maryland, United States; The University of Alabama, Tuscaloosa, Alabama, United States; Cognitive Dynamics Foundation, Tuscaloosa, Alabama, United States

## Abstract

The University of Alabama experiential learning program, Bringing Art to Life (BATL) pairs teams of undergraduate students with persons with dementia attending an adult day services program and engaging in structured art therapy and reminiscence. Graduate students in clinical geropsychology work with an interdisciplinary team and conduct outcomes research focused both on individuals with dementia and student learners. Across seven semesters, we found that students in the BATL course compared with students in either didactic psychology of aging courses or an introductory psychology courses demonstrated improved attitudes towards persons with dementia and increased empathy. Graduate and undergraduate students learned mixed method and community-engaged research methods, producing clinically meaningful research. Experiential learning is essential to the recruitment of a new gerontology workforce.

